# Correction to: Real-world multicentre cohort of first-line pembrolizumab alone or in combination with platinum-based chemotherapy in non-small cell lung cancer PD-L1 ≥ 50%

**DOI:** 10.1007/s00262-023-03405-7

**Published:** 2023-02-27

**Authors:** E. Pons-Tostivint, P. Hulo, V. Guardiolle, L. Bodot, A. Rabeau, M. Porte, S. Hiret, P. Demontrond, H. Curcio, A. Boudoussier, R. Veillon, M. Mayenga, C. Dumenil, T. Chatellier, P. A. Gourraud, J. Mazieres, J. Bennouna

**Affiliations:** 1 Centre Hospitalier Universitaire Nantes, Medical Oncology, Nantes University, 44000 Nantes, France; 2 Medical Oncology Unit, Clinique Mutualiste de L’Estuaire, Saint-Nazaire, France; 3 Centre Hospitalier Universitaire Nantes, Institute of Health and Medical Research, Santé Publique, Clinique Des Données, Inserm CIC 1413, Centre Hospitalier Universitaire de Nantes, Nantes University, 44000 Nantes, France; 4 Thoracic Oncology Department, Hôpital Larrey, CHU Toulouse, 31000 Toulouse, France; 5 Department of Medical Oncology, Comprehensive Cancer Center, Institut de Cancérologie de L’Ouest, Saint-Herblain, France; 6 Department of Pneumology, Centre François Baclesse, Caen, France; 7 Department of Pneumology, University Hospital of Bordeaux, Pessac, France; 8 Department of Medical Oncology, Hospital Foch, Suresnes, France; 9 Department of Respiratory Diseases and Thoracic Oncology, APHP-Hopital Ambroise Pare, 92100 Boulogne-Billancourt, France

Correction to: Cancer Immunology, Immunotherapy https://doi.org/10.1007/s00262-022-03359-2

The article Real‑world multicentre cohort of first‑line pembrolizumab alone or in combination with platinum‑based chemotherapy in non‑small cell lung cancer PD‑L1 ≥ 50%, written by E. Pons‑Tostivint, P. Hulo, V. Guardiolle, L. Bodot, A. Rabeau, M. Porte, S. Hiret, P. Demontrond, H. Curcio, A. Boudoussier, R. Veillon, M. Mayenga, C. Dumenil, T. Chatellier, P. A. Gourraud, J. Mazieres, J. Bennouna, was originally published electronically on the publisher’s internet portal on 24 January 2023 without open access. With the author(s)’ decision to opt for Open Choice the copyright of the article changed on 8 February 2023 to © The Author(s) 2022 and this article is licensed under a Creative Commons Attribution 4.0 International License, which permits use, sharing, adaptation, distribution and reproduction in any medium or format, as long as you give appropriate credit to the original author(s) and the source, provide a link to the Creative Commons licence, and indicate if changes were made. The images or other third party material in this article are included in the article’s Creative Commons licence, unless indicated otherwise in a credit line to the material. If material is not included in the article's Creative Commons licence and your intended use is not permitted by statutory regulation or exceeds the permitted use, you will need to obtain permission directly from the copyright holder. To view a copy of this licence, visit http://creativecommons.org/licenses/by/4.0/.

The original article has been corrected.

